# Double-tract reconstruction for oesofagocardial gastric cancer: A systematic review

**DOI:** 10.1016/j.amsu.2021.102496

**Published:** 2021-06-22

**Authors:** K.V. Stegniy, E.V. Maslyantsev, R.A. Goncharuk, A.A. Krekoten, T.A. Kulakova, E.R. Dvoinikova

**Affiliations:** Department of Surgery, Medical Center of Far Eastern Federal University, 690922, 10 Ajax Bay, Russky Island, Vladivostok, Russia

**Keywords:** Oncology, Gastrectomy, Proximal gastrectomy, Double-tract reconstruction, Gastric cancer, DT, double-tract, PG, proximal gastrectomy, TG, total gastrectomy

## Abstract

The number of people with gastric cardia and distal oesophageal cancers has increased in the last five years. The surgical treatment method of choice is proximal gastrectomy, with an option being reconstruction of the gastrointestinal tract. There are many reconstruction techniques for anastomosis of the oesophagus and distal parts of the digestive tract. However, all can result in complications. This systematic review aims to identify the efficacy of the double-tract reconstruction method after gastric resection. Different operative techniques for gastric reconstruction have been included in this review. The double-tract reconstruction method, which is gaining popularity among surgeons in Asia and Europe, is a promising technique that improves the early and late results of surgical treatment. This method is associated with low complications related to gastroesophageal reflux disease and dysphagia. Double-tract reconstruction is a promising method for the treatment of patients with esofagocardial gastric cancer. However, further studies are required on the long-term complications and side effects.

## Highlights

• For the past 10 years there've been an increase in number of cases of proximal stomach and gastroesophageal junction cancers.
• Double tract reconstruction can restore normal metabolic functions at the remote postoperative period.
• Double tract operation can give the opportunity for free access to the major duodenal papilla by the preservation of gastrointestinal tract continuity.

## 1 Introduction

Gastric cancer is the fifth most common cancer (5.7%) among malignant neoplasms and the third most common cause of cancer-related deaths worldwide (8.3%). Despite the global decline in the number of new cases of non-cardia gastric cancer (approximately 1.5% decrease annually) for several decades, there has been an increase in the number of new cases of proximal stomach and gastroesophageal junction cancers (27% of the total number of gastric cancer cases) [[1], [2], [3]]. In some European and Asian countries, the incidence of gastric cardia cancer is close to or even exceeds the total number of new cases of gastric cancer in other localisations. However, the reasons for this are unknown, and several studies are currently being conducted to examine the reasons for this [4,8]. Potential risk factors for non-cardia gastric cancer are low socioeconomic status (low-quality food), eating habits (intake of a high amount of smoked and spicy food), and the spread of highly virulent *Helicobacter pylori* strains. According to some sources, the prophylaxis and treatment of *H. pylori* can prevent cancer development [4].

The pathogenesis of gastric cardia cancer is not fully understood. However, there are two variants of its development. The first is associated with obesity and gastroesophageal reflux disease and presents as adenocarcinoma of the oesophagus [5]. The second type is associated with superficial gastropathy and *H. pylori*, presenting as cancer in the non-cardia parts of the stomach [5].

*H. pylori* infection is known to cause gastropathy in the stomach, increasing the risk of non-cardia gastric cancer development [6,7]. At the same time, there is evidence that the *H. pylori* bacteria protect against gastroesophageal reflux by lowering the secretion of hydrochloric acid in the proximal stomach, thus lowering the oncologic risk at that localisation [6,7]. However, this has not been thoroughly studied and is the subject of further research [8].

Therefore, the success of treatment against *H. pylori*, which was found to decrease the incidence of non-cardia gastric cancers in developed countries, also increases the occurrence of cardia cancer [6,9].

However, it is impossible to define the exact percentage of cardia gastric cancer among the other locations due to the number of people with late-stage cancer that has spread [[10], [11], [12]].

Surgery is a radical treatment method for this pathology. The major complications after surgery at the site of the gastroesophageal junction are dehiscence of anastomosis, reflux esophagitis in case of anastomosis between the oesophagus and residuary stomach, stenosis at the site of anastomosis, and pyloric stenosis (postvagotomy syndrome) [3,13,14]. It is assumed that double-tract reconstruction (DT) may avoid or significantly decrease the occurrence of these complications and maintain the patient's physiological and nutritional status as well as avoid critical body weight loss in the postoperative period [15,16].

This method involves the replacement of the removed part of the oesophagus and stomach by the isoperistaltic jejunum loop with sequential anastomotic formation between the oesophagus, stomach, and afferent limb of the jejunum [17]. During proximal gastrectomy (PG), the key features of this technique are resection of the oesophagus and stomach with the transection of the jejunum loop at 20–25 cm from the ligament of Treitz with sequential formation of oesophagoenteroanastomosis and gastroenteroanastomosis with the remaining part of the stomach, followed by entero-entero anastomosis for the reconstruction of the small bowel passage [18]. In cases with distal gastric cancer or a high extent, oesophagoenteroanastomosis, duodenoenteroanastomosis, and entero-entero anastomosis are performed after total gastrectomy transection of the isoperistaltic jejunum loop [19].

## 2 Materials and methods

Systematic search of international literature (PubMed, Web of Science, EBSCO, Cochrane Library) and Russian literature (Elibrary.ru, Cyberleninka.ru) the databases were conducted between 2010 and September 2020. The search terms were Double Tract Reconstruction, Proximal Gastric Resection, Complete Gastric Resection, Double Tract. Using the above criteria, a total of 12,430 articles were identified. The analysis included studies that compared different types of reconstruction after PG and total gastric resection (TG), as well as separate systematic reviews and case reports that included DT. Studies involving reports of TG and PG, without discussion of DT, lymphadenectomy, changes in the treatment of gastric cancer, case reports were excluded. A total of 22 articles were identified. Data on the duration of the operation, intraoperative blood loss, postoperative complications (divergence of the anastomosis, reflux esophagitis, stenosis at the site of the anastomosis, gastrostasis), nutritional status and quality of life were extracted and analyzed from these articles.

Work has been reported in line with the PRISMA criteria [20].

### 2.1 Research accordance with AMSTAR 2 [21]

Research registered in Prospero CRD42021237191. https://www.crd.york.ac.uk/prospero/display_record.php?ID=CRD42021237191.

## 3 Results and discussion

PG with DT was generally compared to TG or reconstruction by small bowel part insertion using an open or laparoscopic approach in the included studies.

There were comparable results in all the studies in terms of tumour recurrence, metastasis, and long-term survival in the patients that underwent DT compared to those who underwent TG with Roux-en-Y reconstruction and reconstructive techniques after PG for Siewert type II and III adenocarcinomas [18,22,37].

Formation of esophago-entero and gastro-entero anastomosis for DT could be made using a linear or circular stapler or OrVil™ system. There are studies on all the above techniques and their results [[23], [24], [25]]. However, no studies have compared functional results and correlations between different techniques regarding the number of dehiscences.

### 3.1 Operative time and blood loss

Most studies did not show any statistically significant differences in operative time and intraoperative blood loss in those who underwent PG with DT (PG-DT) and TG for conventional and laparoscopic procedures [19,26,27].

Jung et al. found that operative time and blood loss were lower in those who underwent PG-DT than laparoscopic TG. This may depend on surgical experience and the technique used to create anastomoses [18].

### 3.2 Early and late postoperative complications

Considering postoperative complications such as reflux esophagitis, anastomosis dehiscence, and stenosis, some researchers concluded that there was no significant difference in the rate of complications between DT and TG [28].

A study by Aburatani et al. revealed that the rate of reflux esophagitis (10.5% vs. 54.4%) and stenosis at the site of anastomosis (0% vs. 27%) in those that underwent PG-DT was lower than those that underwent PG with oesophagogastroanastomosis. These results suggest the efficacy of an intestinal insert between the oesophagus and stomach [29].

In a meta-analysis by Shaibu Z. Chen. Z. et al., which included eight studies with 171 patients, the incidence of postoperative complications after DT was 9.6%, 3.5%, 3.9%, and 39.6% for reflux esophagitis, anastomotic stricture, anastomosis dehiscence, and gastrostasis, respectively [30].

In the same review, data from 15 studies were included on using the interposition of the jejunum loop as the reconstruction method. The rates of postoperative complications included reflux esophagitis (13.8%), anastomotic stricture (11.3%), anastomosis dehiscence (4.1%), and gastrostasis (41.5%) [30].

There were nine studies that included the imposition of oesophagogastroanastomosis reconstructive technique, with the following results: reflux esophagitis (19.3%), anastomotic stricture (13.0%), anastomosis dehiscence (4.6%), and gastrostasis (21.8%) [30].

There are conflicting data on the long-term results. In the works of Kim DJ, Kim W et al., and Park et al. the data on the lower requirement of vitamin B12 and iron preparations in the postoperative period in those that underwent DT are presented [[31], [32]]. At the same time, other studies have provided data on the absence of a significant difference between changes in blood test parameters (haemoglobin, ferritin, ferritin saturation, total protein, albumin, and cholesterol) and the development of anaemia in those that underwent DT or TG [33]. For example, although ferritin and haemoglobin levels have been reported to be higher in those that underwent DT, the values of both those that underwent DT or TG were within the normal range. In this case, the researchers also concluded that in the DT group, fewer patients required vitamin B12 supplementation [34]. These results indicate the functionality of the preserved part of the stomach during the digestive process [35].

A long-term decrease in the body mass index (BMI) was more favourable in the DT group in all the studies reviewed. After six months of observation, the decrease in BMI of the group after gastrectomy was 14.9% and 5.7% after PG-DT. After 12 months, the changes were 17.9% and 9.6%, respectively [36].

In a study by Nam-ryong Choi et al., 37 patients who underwent laparoscopic PG-DT reconstruction were examined. The authors concluded that maximum weight loss was observed one year after surgery (6.1%). After that, patients' body weights gradually increased. Furthermore, after three years of recovery, patients’ body weights were 96.8% of the preoperative level. The haemoglobin level in the blood serum decreased the most (by 5.9%) one year after surgery and then gradually increased. After two years, the level was higher than before surgery. In addition, the serum iron level in patients increased after surgery and was at its maximum after two years. Vitamin B12 in the blood reached a minimum after six months and then fluctuated. Moreover, the level of albumin was higher than the preoperative level six months after surgery [37].

## 4 Conclusions

The main disadvantages of the DT method are reflux esophagitis and obstruction of the passage of food through the rest of the stomach [27,30,38]. This could be due to the presence of pyloric spasms from postvagotomy syndrome. Reflux esophagitis may occur due to the reconstruction and surgical techniques used during anastomosis. For example, the length of the small intestine section being incorrectly chosen when forming an insert between the oesophagus and the stomach.

The results of this review indicate the need for further study of DT to determine its efficacy. It is also necessary to search for a solution that limits the disadvantages to this reconstruction technique (improvement of the operative technique, acceptable anastomosis techniques, and vagus-sparing surgical intervention methods).

Given the lack of reliable data on the development of postoperative complications and the advantages of this method over other interventions, DT is a promising technique for surgical intervention in pathology of the cardioesophageal junction. However, further studies in experimental and clinical conditions are required.

## Ethical approval

All procedures performed in this study involving human participants were in accordance with the ethical standards of the institutional and/or national research committee and with the 1964 Helsinki Declaration and its later amendments or comparable ethical standards.

## Sources of funding

Non-commercialized scientific review article. No funding from any source.

## Author contribution

K.V. Stegniy: study concept, data interpretation, writing the paper.

E.V. Maslyantsev: study concept, data interpretation, writing the paper.

R.A. Goncharuk: study concept, data interpretation, writing the paper.

A.A. Krekoten’: data collection.

T.A. Kulakova: data collection.

E.R. Dvoinikova: data collection.

## Research registration number

Name of the registry: PROSPERO

Unique Identifying number or registration ID: CRD42021237191

Hyperlink to your specific registration (must be publicly accessible and will be checked):https://www.crd.york.ac.uk/prospero/display_record.php?ID=CRD42021237191

## Guarantor

K.V. Stegniy.

## Provenance and peer review

Not commissioned, externally peer-reviewed.

## Declaration of competing interest

None.
